# Integrating a dual-silicon photoelectrochemical cell into a redox flow battery for unassisted photocharging

**DOI:** 10.1038/ncomms11474

**Published:** 2016-05-04

**Authors:** Shichao Liao, Xu Zong, Brian Seger, Thomas Pedersen, Tingting Yao, Chunmei Ding, Jingying Shi, Jian Chen, Can Li

**Affiliations:** 1 State Key Laboratory of Catalysis, Dalian Institute of Chemical Physics, Chinese Academy of Sciences, Dalian National Laboratory for Clean Energy, iChEM, Dalian 116023, China; 2 University of Chinese Academy of Sciences, Beijing 100049, China; 3 Department of Physics, CINF, Technical University of Denmark, Kongens Lyngby 2800, Denmark; 4 Department of Micro- and Nanotechnology, Technical University of Denmark, Kongens Lyngby 2800, Denmark

## Abstract

Solar rechargeable flow cells (SRFCs) provide an attractive approach for *in situ* capture and storage of intermittent solar energy via photoelectrochemical regeneration of discharged redox species for electricity generation. However, overall SFRC performance is restricted by inefficient photoelectrochemical reactions. Here we report an efficient SRFC based on a dual-silicon photoelectrochemical cell and a quinone/bromine redox flow battery for *in situ* solar energy conversion and storage. Using narrow bandgap silicon for efficient photon collection and fast redox couples for rapid interface charge injection, our device shows an optimal solar-to-chemical conversion efficiency of ∼5.9% and an overall photon–chemical–electricity energy conversion efficiency of ∼3.2%, which, to our knowledge, outperforms previously reported SRFCs. The proposed SRFC can be self-photocharged to 0.8 V and delivers a discharge capacity of 730 mAh l^−1^. Our work may guide future designs for highly efficient solar rechargeable devices.

## Abstract (web summary)

Technologies for *in situ* capture and storage of intermittent solar energy are an important research goal. Here the authors report a solar rechargeable flow cell based on a dual-silicon photoelectrochemical cell and a quinone/bromine redox flow battery for *in situ* solar energy conversion and storage.

## Graphical abstract

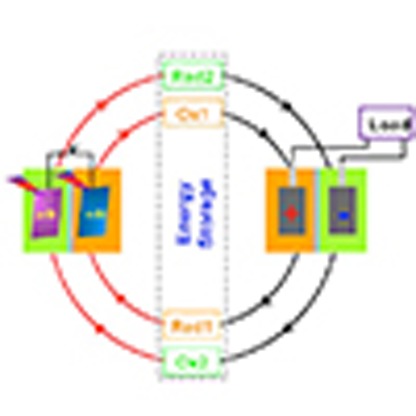

Simultaneous conversion and storage of abundant, but intermittent solar energy has been entering the spotlight as a promising strategy for the controllable utilization of solar energy^1^,^2^. Specifically, directly storing solar energy in hydrogen (H_2_) produced by light-driven water splitting has been regarded particularly attractive, as hydrogen is an efficient and clean fuel for electricity generation^3^,^4^. Unfortunately, challenges with hydrogen storage and the cost of fuel cells impede wide implementation of solar hydrogen/fuel cell hybrid systems. Moreover, the sluggish half reaction kinetics of water oxidation greatly hamper the improvement of solar energy conversion efficiency in water splitting^5^,^6^.

Alternatively, solar energy can be *in situ* stored in other chemicals by driving non-spontaneous reactions in a photoelectrochemical (PEC) cell^7^. The resulting products can be readily utilized to generate electricity via reversible chemical reactions. On the basis of this principle, efforts to fabricate solar rechargeable cells (SRCs) have been going on for several decades^8^. For instance, solid state electrodes can be integrated in SRCs for storing photogenerated charges^9^,^10^,^11^,^12^. However, the storage capacity of SRCs is limited by the physical dimension of solid electrodes. Besides, the sluggish insertion/extraction of the ions into/from the solid electrodes may lead to a poor energy conversion efficiency. This has prompted efforts to integrate SRCs and redox flow batteries (RFBs) with two soluble redox species to improve the storage capacity^13^. Redox couples in RFBs generally present facile electrochemical kinetics, which can be several orders of magnitudes faster than that of water oxidation^14^. Benefiting from the rapid semiconductor/electrolyte interface charge transfer, a higher solar-to-chemical (STC) conversion efficiency based on fast redox species could be expected in comparison with that of solar to hydrogen in water splitting. A conceptual SRC with continuous flow electrolytes was reported by Yang *et al*., but the overall energy conversion efficiency (<0.1%) is limited by the low conductivity of the inorganic separator between the aqueous and organic electrolytes^15^,^16^. More recently, Liu *et al*., proposed to photocharge a vanadium RFB using wide bandgap semiconductors such as WO_3_ and TiO_2_^17^,^18^. However, the STC conversion efficiency was low and the discharge performance has not yet been reported. Fabricating an aqueous SRC with high STC conversion efficiency and desirable storage capacity still remains a challenge.

Here we demonstrate a solar rechargeable flow cell (SRFC) integrating a dual-silicon PEC cell into a quinone/bromine RFB for *in situ* solar energy conversion and storage. To attain significant improvements in SRC performance, two important factors in the PEC reactions guide our design. First, a photoelectrode with a narrow bandgap is required to allow for efficient utilization of solar energy. Second, redox couples with fast reaction kinetics and efficient cocatalysts that can catalyse the redox reactions should be employed to expedite semiconductor/electrolyte interface charge transfer kinetics. In this regard, silicon with a favourable bandgap (1.1 eV) is used as the light absorber. Quinones and halogens, which present fast reaction kinetics and excellent electrochemical reversibility^19^,^20^,^21^, are used as energy storage media for efficiently capturing photogenerated charges. In our SRFC, the fast PEC reactions of the water-soluble quinone and bromine redox couples on the buried junction and cocatalyst-functionalized dual-silicon absorbers enable the SRFC to achieve an overall photon–chemical–electricity energy conversion efficiency of ∼3.2% and deliver a constant discharge voltage of ∼0.78 V, which, to our knowledge, are higher than values achieved previously in SRFCs^15^,^16^,^17^,^18^. Our work demonstrates aqueous SRFCs with good overall efficiency, high discharge voltage and desirable discharge capacity, and suggests paths for further improvements to allow technological development and use.

## Results

### Configuration and working principle of the SRFC

**Figure 1:**
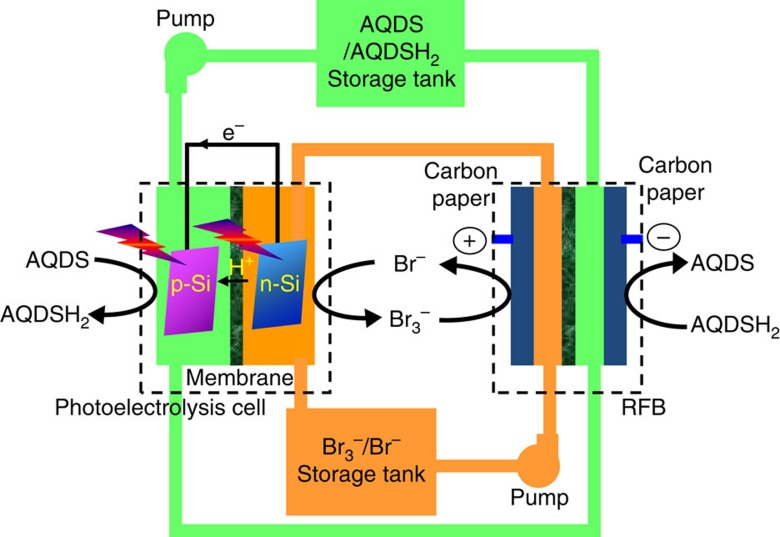
Schematic configuration of the proposed SRFC. AQDS/AQDSH_2_ and Br_3_^−^/Br^−^ are used as redox couples.

Figure 1 illustrates the configuration of the SRFC used in this work. It consists of a PEC (or photoelectrolysis) cell that deposits solar irradiation into chemical energy and a RFB that converts the as-stored chemical energy into electricity. AQDS/AQDSH_2_ (9,10-anthraquinone-2,7-disulphonic sodium/1,8-dihydroxy-9,10-anthraquinone-2,7-disulphonic sodium) and Br_3_^−^/Br^−^ are used as active redox couples. The PEC cell and RFB are connected through electrolyte circuit loops. During the photocharge process, AQDS is reduced to AQDSH_2_ on the photocathode and Br^−^ is oxidized to Br_3_^−^ on the photoanode simultaneously in the PEC cell by short-circuiting the two photoelectrodes under illumination. The resultant AQDSH_2_ and Br_3_^−^ are then stored in two individual reservoirs that can be readily used by the RFB. A commercial Nafion membrane is used to separate the two compartments in each cell. The involved cell reactions can be expressed as follows:


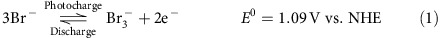



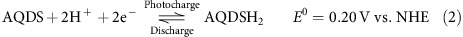


The overall reaction:


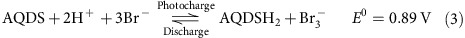


Energy conversion in this SRFC clearly follows a two-step route of solar-chemical-electricity. The overall efficiency is thus decided by the product of the STC efficiency in PEC cell and the chemical-to-electricity efficiency in RFB (*η*_overall_=*η*_STC_ × *η*_RFB_). The quinone/bromine flow battery generally demonstrates a high energy efficiency of over 70% (ref. 19). In this way, the STC efficiency during the photocharging process is a critical issue for the as-designed SRFC. Compared with single-photoelectrode system, dual-photoelectrode configuration has the merits of using small bandgap semiconductor materials that allow for efficient light harvesting and two semiconductor/electrolyte liquid junctions for sufficient photovoltage. Crystal silicon, with a small bandgap of 1.1 eV, is a promising material for STC conversion in a p/n dual-electrode PEC cell^22^,^23^. But so far, its applications are limited due to the instability^24^, sluggish surface charge transfer kinetics^25^ and insufficient self-driving force^26^. Recent efforts show that the sustainability of crystal silicon in water splitting reaction can be significantly improved to tens of hours by surface passivation and/or cocatalyst^27^,^28^,^29^,^30^,^31^,^32^,^33^. Moreover, fabrication of a surface-buried junction (n^+^p or p^+^n) generally induces a photovoltage of ∼0.50 V (refs 22, 23, 34). Thereby, the dual-silicon (n^+^p-Si and p^+^n-Si) PEC system is able to offer a photovoltage up to 1.0 V, which is thermodynamically sufficient to drive the above overall reaction for photocharging without the assistance of external bias.

### Reduction half reaction during photocharging process

**Figure 2:**
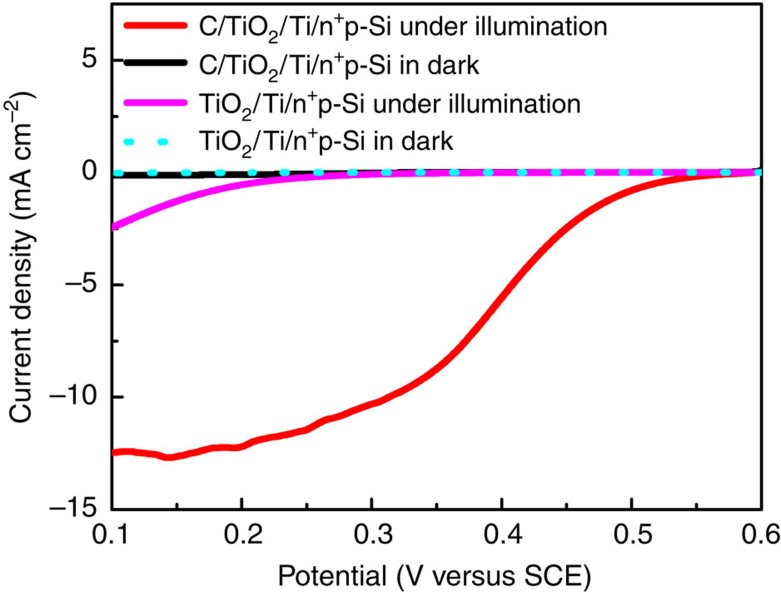
Photoelectrochemical reduction of AQDS over photocathode. Current–potential curves of the photocathodes in a 0.05 M AQDS+1.0 M H_2_SO_4_ solution purged with argon under AM 1.5-G 100 mW cm^−2^ illumination.

According to the working principle, photocharging of a SRFC involves the PEC reduction of AQDS and PEC oxidation of Br^−^. As a proof-of-concept study, PEC reduction of AQDS to produce discharging species of AQDSH_2_ is firstly studied. The n^+^p-Si coated orderly with metal titanium and TiO_2_ thin films as a protective layer (hereafter TiO_2_/Ti/n^+^p-Si) is employed as the photocathode. It is reported that the carbon material serves as an excellent substrate for adsorption and electron transfer for the quinone/hydroquinone redox reaction^35^. Therefore, a carbon film of 5-nm thickness is sputtered onto the TiO_2_/Ti/n^+^p-Si electrode to achieve a carbon covered surface (hereafter C/TiO_2_/Ti/n^+^p-Si), as evidenced by the scanning electron microscopy images and X-ray photoemission spectroscopy analysis (Supplementary Fig. 1a,c). Figure 2 shows the current–potential (*I*–*E*) curves of TiO_2_/Ti/n^+^p-Si and C/TiO_2_/Ti/n^+^p-Si electrodes in the electrolyte containing 0.05 M AQDS. Both electrodes generate insignificant currents in the dark. While under illumination, the TiO_2_/Ti/n^+^p-Si photocathode produces a photocurrent density of −2.5 mA cm^−2^ at 0.1 V versus SCE (the saturated calomel electrode) and has an onset potential of 0.25 V versus SCE (defined as the potential at which a photocurrent exceeds 0.2 mA cm^−2^). Upon carbon modification, the photocurrent density significantly increases to −12.5 mA cm^−2^ at the same bias. Meanwhile, the onset potential experiences a positive shift of ∼300 mV. These results suggest that carbon is a more favourable substrate for the PEC reduction of AQDS than TiO_2_. In sharp contrast, the currents from C/TiO_2_/Ti/n^+^p-Si photocathodes, both in the dark and under illumination, are negligible in the absence of AQDS (Supplementary Fig. 2a). Thus, the observed photocurrents can be almost completely attributed to AQDS reduction. Since photocurrent spikes grow remarkably as applied potential is decreased under chopped light (Supplementary Fig. 3; Supplementary Note 1), the demonstrated photocurrent is speculated to be limited by the mass transfer of AQDS from bulk solution to the electrode surface.

**Figure 3:**
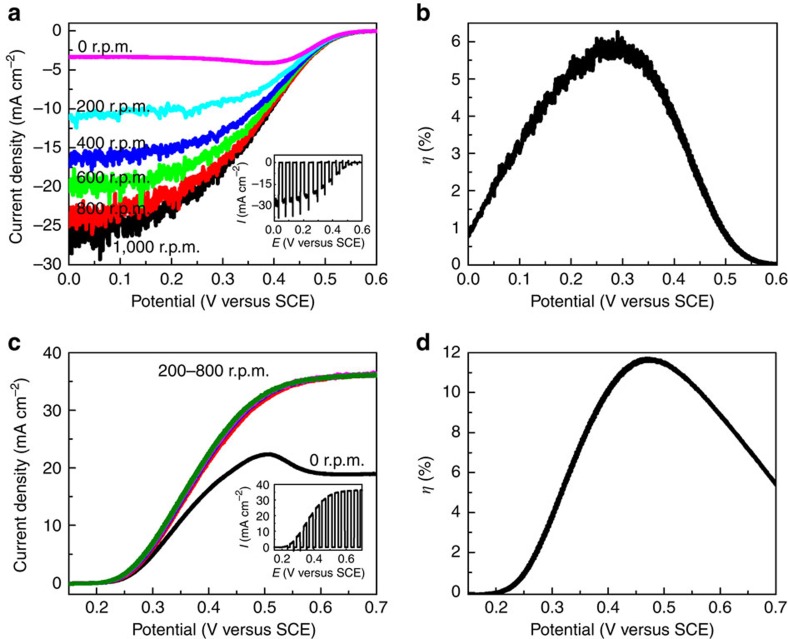
Photoelectrochemical performance of the photoelectrodes. Constant light current–potential curves of the C/TiO_2_/Ti/n^+^p-Si photocathode in a 0.05 M AQDS+1.0 M H_2_SO_4_ solution (**a**) and Pt/p^+^n-Si photoanode in a 0.2 M HBr+1.0 M H_2_SO_4_ solution (**c**) under various magnetically stirring speeds. The inset in **a** and **c** shows chopped light current–potential curves of the photocathode at 1,000 r.p.m. magnetic stirring speed and the photoanode at 200 r.p.m. magnetic stirring speed, respectively. Corresponding half-cell STC conversion efficiency for C/TiO_2_/Ti/n^+^p-Si photocathode at 1,000 r.p.m. stirring speed (**b**) and for Pt/p^+^n-Si photoanode at 200 r.p.m. stirring speed (**d**). Light source: AM 1.5-G 100 mW cm^−2^.

To investigate the mass transfer effect, controlled experiments were performed in magnetically stirred solution at various speeds and the obtained *I*–*E* curves are presented in Fig. 3a. A limiting photocurrent appears on each curve and is enhanced with increasing stirring speeds from 0 to 1,000 r.p.m. At 1,000 r.p.m., the saturated photocurrent reaches −27.0 mA cm^−2^, which is about six times higher than that obtained in static electrolyte. The mass transfer limitation is thus confirmed during the AQDS reduction reaction. Actually, the diffusion limitation cannot be fully avoided even at a stirring speed up to 1,000 r.p.m., which is evidenced by the transient current spikes under chopped light as indicated in the inset of Fig. 3a.

To understand the PEC behaviours of the AQDS species, we measured the kinetic parameter for the electrochemical reduction reaction of AQDS to AQDSH_2_, as well as the diffusion coefficient of AQDS species in the electrolyte via a linear sweep voltammetry method (see Supplementary Fig. 4 and Supplementary Methods for details). The diffusion coefficient (*D*) of AQDS is determined to be 4.8 × 10^−6^ cm^2^ s^−1^, which is in agreement with the previous report^19^. The rate constant (*k*_0_) is evaluated to be 1.05 × 10^−2^ cm s^−1^. Compared with other redox couples employed in RFBs such as Fe^3+^/Fe^2+^, Cr^3+^/Cr^2+^, Ce^4+^/Ce^3+^, VO_2_^+^/VO^2+^ and V^3+^/V^2+^ (Supplementary Table 1), the AQDS/AQDSH_2_ on carbon electrode exhibits much larger *k*_0_ in combination with a comparable diffusion coefficient. The extremely rapid kinetics might be attributed to little reorganizational energy required in the electrochemical reduction of anthraquinone^20^. However, the transport of AQDS species in the electrolyte is too slow to keep up with the prompt interface charge transfer. These electrochemical properties result in the dominance of mass transfer where it is particularly prominent at more negative applied potentials, which well explains the unusual PEC behaviour of AQDS on C/TiO_2_/Ti/n^+^p-Si photocathode. The reversible potential for AQDS over a graphite electrode is evaluated to be −0.03 V versus SCE (Supplementary Fig. 2b). On the basis of equation (4) presented below^36^, the maximum solar-to-AQDSH_2_ conversion efficiency can be calculated to be ∼6.0% at a bias of 0.30 V versus SCE (Fig. 3b).

### Oxidation half reaction during photocharging process

We then moved on to investigate the PEC oxidation of Br^−^ to Br_3_^−^, which is the other STC reaction during the storage of solar energy into chemical energy. The p^+^n-Si coated with platinum islands as cocatalyst (scanning electron microscopy and X-ray photoemission spectroscopy, respectively, in Supplementary Fig. 1b,d, hereafter Pt/p^+^n-Si) is employed as the photoanode. Figure 3c demonstrates the *I*–*E* curves over Pt/p^+^n-Si photoanode in the solution containing 0.2 M HBr and 1.0 M H_2_SO_4_. Without stirring the electrolyte, the photocurrent is observed to reach a peak value of 22.2 mA cm^−2^ at 0.50 V versus SCE and beyond which decreases to a constant value of 19.0 mA cm^−2^. In comparison, control experiments show that the currents are negligible without the presence of Br^−^ species both under light and in the dark (Supplementary Fig. 5a). Therefore, the photocurrent generated is ascribed to the photo-oxidation of Br^−^. With magnetic stirring of the solution at 200 r.p.m., the current peak vanishes and the limiting photocurrent promptly elevates to a much higher level of 36.2 mA cm^−2^. Unlike the electrochemical behaviour of AQDS during the PEC reduction, increase of the stirring speed to 400–800 r.p.m. does not induce further improvement in the saturated photocurrent. Moreover, no transient current spikes under chopped light irradiation are observed even at more positive bias, indicating that most photogenerated holes arriving at the electrode/electrolyte interface participate in the oxidation reaction (inset in Fig. 3c). It is worth noting that the saturation photocurrents in stirred solution are completely dominated by the incident light intensity (Supplementary Fig. 6a,b) and the mass transfer limitation during the PEC oxidation of Br^−^ thus is fully relieved, which is different from the behaviour of AQDS reduction.

The *k*_0_ for the Br^−^ oxidation reaction and the *D* for Br^−^ ions were also determined (Supplementary Fig. 7) and listed in Supplementary Table 1. Compared with those parameters relative to AQDS, the *k*_0_ (2.82 × 10^−2^ cm s^−1^, which is consistent with existing kinetics data^37^) is of the same order while *D* (6.0 × 10^−5^ cm^2^ s^−1^) increases by one order of magnitude. The large diffusion coefficient of Br^−^ allows us to sidestep mass transfer limitations during PEC oxidation reactions. It is also noteworthy that the *k*_0_ for Br^−^ oxidation is more than twice the value for AQDS reduction and much larger than values for the other redox couples listed in Supplementary Table 1. This ensures rapid charge transfer between the photoelectrode and soluble species, resulting in more effective utilization of the photogenerated carriers. The larger saturated photocurrent is achieved in the case of Br^−^ oxidation than AQDS reduction (Fig. 3a,c). The reversible potential for Br_3_^−^/Br^−^ is estimated to be 0.85 V versus SCE on a Pt electrode (Supplementary Fig. 5b). According to equation (4), the optimal half-cell solar-to-Br_3_^−^ conversion efficiencies is ∼11.6% at 0.46 V versus SCE as indicated in Fig. 3d, which is nearly two times as large as that of solar to AQDSH_2_. In addition, the maximal power conversion efficiency of Pt/p^+^n-Si photoelectrode for the electrochemical photovoltaic cell approaches 12.0% (Supplementary Fig. 8), slightly higher than the previously reported value of 10.5% (ref. 38). The high efficiency further confirms the rapid electrochemical kinetics of Br^−^ oxidation.

### Performance and stability assessment of the PEC cell

**Figure 4:**
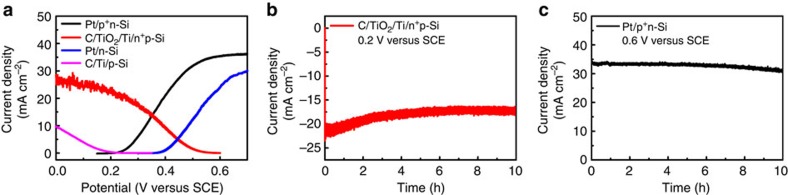
Photoelectrochemical behaviours of various photoelectrodes. (**a**) Overlaid current–potential curves of the individual photocathodes and photoanodes measured in a three-electrode experiment. C/Ti/p-Si and C/TiO_2_/Ti/n^+^p-Si in a 0.05 M AQDS+1.0 M H_2_SO_4_ solution; while Pt/n-Si and Pt/p^+^n-Si/ in a 0.2 M HBr+1.0 M H_2_SO_4_ solution. Chronoamperometry of the C/TiO_2_/Ti/n^+^p-Si photocathode in a 0.05 M AQDS+1.0 M H_2_SO_4_ solution (**b**) and the Pt/p^+^n-Si photoanode in a 0.2 M HBr+1.0 M H_2_SO_4_ solution (**c**). The applied stirring speeds were 1,000 r.p.m. in the AQDS solution and 700 r.p.m. in the HBr solution, respectively. Light source: AM 1.5-G 100 mW cm^−2^.

After investigating the photoelectrodes in the corresponding electrolytes independently, we can evaluate the performance of the integrated photoanode/cathode PEC cell by overlapping the individual *I*–*E* data for each photoelectrode as shown in Fig. 4a. The intersection of the two curves indicates the optimal operating current density and the overall STC (*η*_O-STC_) efficiency in the resulted PEC cell can be calculated according to equation (5) (ref. 5). It can be observed that the intersection is located at 13.5 mA cm^−2^ at 0.35 V versus SCE and the corresponding *η*_O-STC_ is estimated to be 5.9% for the proposed dual-silicon PEC cell, which is much higher than those of direct solar water splitting, as well as all the reported SRFCs^15^,^16^,^17^,^18^,^39^. Considering that the energy conversion efficiency in this quinone/bromine flow battery is *∼*70% (Supplementary Fig. 9), the overall energy conversion efficiency of the present SRFC is estimated to be 4.1% (*η*_overall_=*η*_O-STC_ × *η*_RFB_=5.9 × 70%). It should be pointed out that no bias is required to drive the overall photocharge reactions. For comparison, the *I*–*E* curves for the silicon wafers without a surface doping, C/Ti/p-Si and Pt/n-Si photoelectrodes, are also listed in Fig. 4a. No intersection between these two curves can be observed, indicating that the photocharge reaction in the C/Ti/p-Si∥Pt/n-Si dual-photoelectrode system cannot be realized without additional power input. For the C/Ti/p-Si∥Pt/p^+^n-Si and C/TiO_2_/Ti/n^+^p-Si∥Pt/n-Si photoelectrolysis cells, the operating photocurrents during solar-charge process are rather low and thus the overall energy conversion efficiencies are far from satisfactory.

Current–time (*I*–*t*) tests reveal a photocurrent of ∼13 mA cm^−2^ for each photoelectrode at the operating potential of 0.35 V versus SCE (Supplementary Fig. 10a,c), which agree with the data obtained from the above *I*–*E* measurements. This result further confirms the predicted STC efficiency as above. Stability tests illustrate that both photoanode and photocathode can sustain much higher currents for at least 10 h under AM 1.5-G irradiation (Fig. 4b,c). The light absorbance of the catholyte changed after PEC reaction (Supplementary Fig. 11a), suggesting the generation of new species and inevitable absorption losses as a result. Therefore, the appropriate concentration of electrolytes is selected for PEC and SRFC demonstration. To investigate photochemical stability of the electrolytes, experiments were conducted under simulated solar irradiation and visible light illumination (Supplementary Fig. 11b–e). The results show that AQDS is stable under visible light, but it undergoes degradation under ultraviolet irradiation. Br^−^ is highly stable under the full solar spectrum. When exposed only to visible light to reduce potential photochemical side reactions, the photocathode can run steadily for >100 h and the photoanode for 37 h (Supplementary Fig. 10b,d). The Faradaic efficiencies for photogenerated AQDSH_2_ and Br_3_^−^ species at the operating potential (0.35 V versus SCE) under visible light illumination are 97% and 99%, respectively (Supplementary Fig. 12; Supplementary Methods), suggesting that the observed photocurrents can be ascribed to the PEC reactions of the electroactive species with negligible side reactions.

### Demonstration of a proof-of-concept SRFC

**Figure 5:**
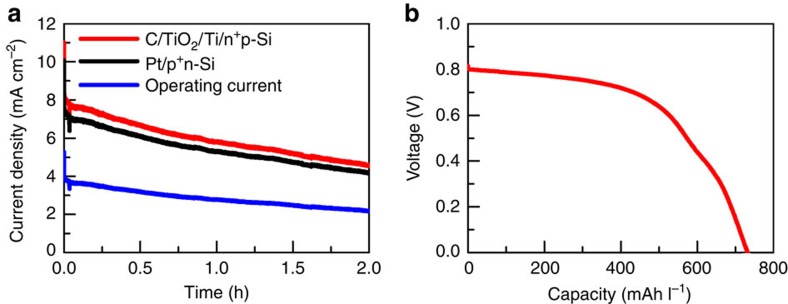
Electrochemical characterizations of the proposed SRFC. (**a**) Photocharge curves of the SRFC in a 0.2 M HBr+1.0 M H_2_SO_4_ solution on the positive side and a 0.05 M AQDS+1.0 M H_2_SO_4_ solution on the negative side without the external bias under AM 1.5-G 100 mW cm^−2^ illumination. The surface area of C/TiO_2_/Ti/n^+^p-Si is 0.232 cm^2^ and that of Pt/p^+^n-Si is 0.254 cm^2^. The flow rates of the electrolytes are kept at 15 ml min^−1^. (**b**) The discharge curve of the SRFC at constant current 0.5 mA cm^−2^ under dark conditions.

Given the desirable individual performance of both PEC cell and RFB (cycling stability of the RFB is described in Supplementary Fig. 9 and Supplementary Note 2), a prototype SRFC device based on a side-by-side dual-silicon photoelectrode system and the mentioned RFB with dual-carbon discharge electrode is fabricated (for experimental details see Supplementary Methods) as shown in Supplementary Fig. 13. During the photocharge process under AM 1.5-G irradiation, the C/TiO_2_/Ti/n^+^p-Si photocathode and the Pt/p^+^n-Si photoanode are directly short-circuited without external bias. Figure 5a illustrates the photocharge profiles for the first 2 h. The charging photocurrent for the photocathode starts at ∼8.2 mA cm^−2^ and decreases to 4.6 mA cm^−2^; the corresponding initial and final values for the photoanode are 7.5 and 4.2 mA cm^−2^. The unequal photocurrent density between the two photoelectrodes is due to a small difference in working area of the electrodes studied herein. The colour of the catholyte in the PEC cell gradually changes from light orange to dark green while that of the anolyte simultaneously varies from colourless to yellow, indicating storage of solar energy. The declined photocurrent during the photocharge is primarily ascribed to the two points: (1) decreased light intensity at the silicon photoelectrode due to the generation of colourful AQDSH_2_ and Br_3_^−^ species in the electrolyte; and (2) the shifts in Nernst redox potential of the redox couples caused by varied concentration of active species. On the bais of the total exposure areas of photoanode and photocathode, the actual output photocurrent density for the complete photoelectrolysis cell varies from 3.9 to 2.2 mA cm^−2^, which is lower than the highest photocurrent density 6.8 mA cm^−2^ (half of 13.5 mA cm^−2^) estimated from the Fig. 4a. Such a deviation may be explained by the inefficient mass transfer of electroactive species in the as-fabricated model system, which is limited by the flow rate of the electrolyte. According to equation (5), the overall STC conversion efficiency during the photocharge process initially achieves 3.4% followed by decrease to the final value of 1.9% (Supplementary Fig. 14). The average STC conversion efficiency is calculated to be 2.5% based on equation (6).

Figure 5b shows the galvanostatic discharge curve of the SRFC performed at 0.5 mA cm^−2^. The initial discharge voltage exceeds 0.80 V and a constant discharge voltage plateau locates at around 0.78 V. It is notable that the discharge capacity is up to 730 mAh l^−1^. To the best of our knowledge, a combination of such values has not been attained previously by integrated SRFCs. After photocharging, this SRFC can drive an 8 mW electric fan (Supplementary Movie 1). The overall solar–chemical–electricity energy conversion efficiency is calculated to be 1.0% according to equation (7), which is less than half of the above average STC conversion efficiency of 2.5%. This may be ascribed to concentration polarization resulted from the slow flow rate of the electrolytes, evaporation of bromine, potential side reaction of AQDS, partial retention of the electroactive species in the electrolytes after discharge, electrolytes leakage from the pumping system and other parasitic energy loss processes.

**Figure 6:**
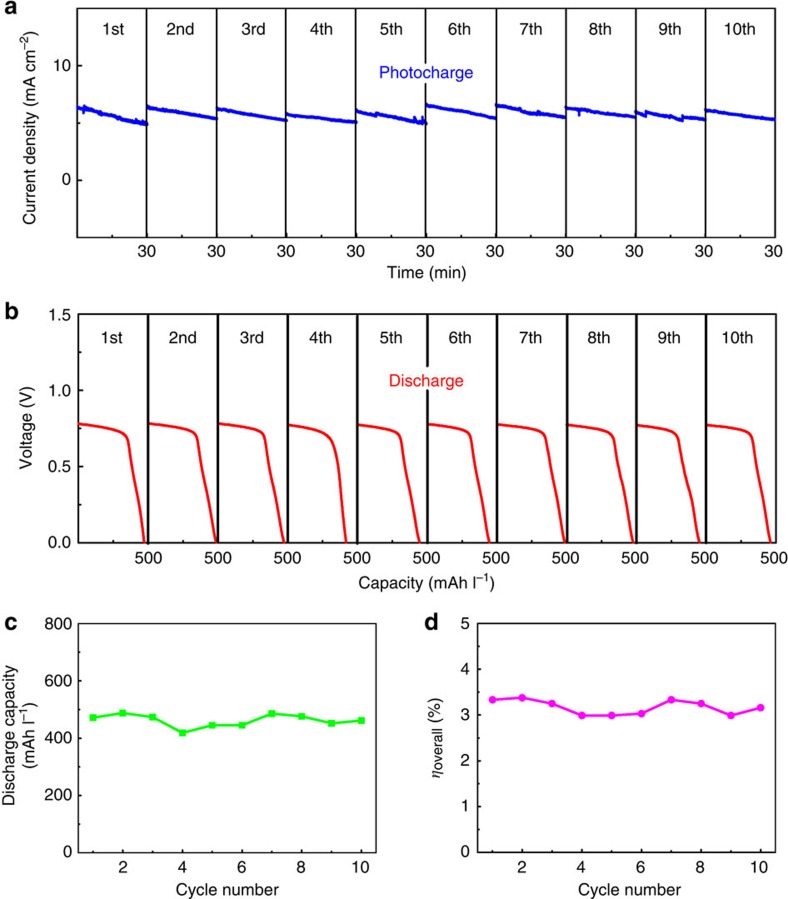
Cycling performance of the proposed SRFC. (**a**) Photocharge curves of the SRFC in a 0.2 M HBr+0.005 M Br_2_+1.0 M H_2_SO_4_ solution on the positive side and a 0.05 M AQDS+1.0 M H_2_SO_4_ solution on the negative side without the external bias under visible light illumination (100 mW cm^−2^). The surface area of C/TiO_2_/Ti/n^+^p-Si is 0.232 cm^2^ and that of Pt/p^+^n-Si is 0.230 cm^2^. The photocurrent density is calculated based on the total illumination areas of photoanode and photocathode. The flow rates of the electrolytes are kept at 100 ml min^−1^. (**b**) Galvanostatic discharge curves of the SRFC at constant current 2.0 mA cm^−2^ under dark. (**c**) Discharge capacity and (**d**) overall photon–chemical–electricity energy conversion efficiency of the SRFC as a function of the cycle number.

While the above SRFC device demonstrates capture and storage of solar energy, its overall solar energy conversion efficiency is far from satisfactory. To address this issue, the device was optimized and photocharged under visible light with a higher flow rate of electrolytes (100 ml min^−1^). In addition, a very small amount of liquid bromine (0.005 M) was added beforehand to the positive electrolyte to replenish the evaporation loss during operation. The repeated photocharge/discharge curves of the SRFC for the first 10 cycles are exhibited in Fig. 6a,b (voltage curves during photocharge shown in Supplementary Fig. 15), respectively. During photocharge, the voltage approaches 0.8 V and the maximum operating photocurrent density is 6.6 mA cm^−2^, which is close to the predicted value (6.8 mA cm^−2^). Almost identical photocharge/discharge curves are presented in the first 10 cycles, implying excellent cycling stability. The SRFC delivers an initial discharge capacity of 471 mAh l^−1^ and maintains a discharge capacity of 462 mAh l^−1^ after 10 photocharge/discharge cycles (Fig. 6c), confirming stable operation. As shown in Fig. 6d, the SRFC possesses highly stable overall photon–chemical–electricity energy conversion efficiency during the 10 photocharge/discharge cycles, with an average efficiency of 3.2%, which to the best of our knowledge is a record-high efficiency attained for the SRFC to date.

## Discussion

The SRFC demonstrated in this work exhibits a number of advantages as follows: first, mainly earth-abundant and eco-friendly materials of silicon and carbon are used as photocharge and discharge electrodes, respectively. Second, in comparison to nonaqueous media, the aqueous electrolyte is safer, cheaper and has higher ionic conductivity. Third, the fast electrochemical kinetics of the selected redox couples not only increases the photocharge efficiency, but also improves the discharged power density of the SRFC. Finally, the SRFC can be flexibly designed and optimized by investigating the PEC cell and RFB individually. On the basis of our design, the photocharge and discharge processes can be performed in the same glass-window equipped cell by immersing the photoelectrodes and electrodes into the corresponding half-cell to fabricate a SRFC with a more compact structure. More efficient SRFCs will be enabled by a combination of the following: optimizing the concentration of electroactive species to keep a balance between photocharge capacity and light absorption of the electrolytes; reducing the distance between the photoeletrode and the light incident window; using the photoelectrode as the light incident window that the irradiation can reach the photoabsorber directly without passing through the electrolyte; applying a glass with TiO_2_ coating or other ultraviolet-absorption materials as the incident window to filter ultraviolet radiation; and developing more transparent redox couples with larger *k*_0_ and *D*, and optimizing device fabrication techniques. Investigation into these approaches is underway in our laboratory.

In summary, we successfully designed and fabricated an aqueous SRFC device with the dual function of conversion and storage of solar energy via direct integration of a dual-silicon PEC cell and a RFB using water-soluble AQDS/AQDSH_2_ and Br_3_^−^/Br^−^ redox couples as the energy carriers. The half-cell STC (H-STC) conversion efficiencies achieved for AQDS reduction on C/TiO_2_/Ti/n^+^p-Si and Br^−^ oxidation on Pt/p^+^n-Si are up to ∼6.0% and ∼11.6%, respectively. The excellent PEC performance can be attributed to the outstanding light harvesting properties of silicon, active surface cocatalysts, as well as rapid kinetics of redox couples. The maximum STC conversion efficiency during photocharge reaches ∼5.9% and the overall photon–chemical–electricity energy conversion efficiency of the as-designed SRFC exceeds 3.0%, record values for an integrated SRFC to the best of our knowledge. In a proof-of-concept SRFC device, the cell voltage can be self-charged to 0.8 V under simulated AM 1.5-G illumination and the discharge capacity is up to 730 mAh l^−1^ after photocharging for 2 h. This aqueous SRFC concept, based on earth-abundant electrodes and fast redox couples, may open new avenues for design and fabrication of highly efficient solar rechargeable batteries towards practical application.

## Methods

### Electrodes preparation

Commercial p-Si wafers were processed to achieve a thin n^+^-doped surface layer followed by successive deposition of 5 nm Ti and 100 nm TiO_2_ protective layers according to reported procedures^22^. A thin carbon layer was then deposited onto the surface of wafers by reactive magnetron sputtering of a high-purity graphite target (99.999%). Further experimental details are provided in the Supplementary Methods.

Commercial n-Si wafers were functionalized by surface p^+^ doping according to a reported procedure and used as photoanodes^23^. Pt was then sputtered on the surface of wafers as a cocatalyst.

Commercial Toray carbon paper electrodes (200 μm in thickness) were used as the positive and negative electrodes in the SRFC system. The carbon papers were first cleaned under sonication in isopropyl alcohol for 10 min, followed by soaking in a 3:1 (by volume) solution of H_2_SO_4_ (conc.) and HNO_3_ (conc.) for 5 h at 323 K.

### Photoelectrochemical measurements

PEC measurements were carried out in a three-electrode system consisting of a photoelectrode as the working electrode, a SCE as the reference electrode and a graphite plate (or platinum plate) as the counter electrode. 1 M H_2_SO_4_ aqueous solutions were used as supporting or blank electrolytes. An aqueous solution containing 0.05 M AQDS (TCI, 95%) and 1.0 M H_2_SO_4_ (Sinopharm Chemical, 98%) was used as the catholyte, and an aqueous solution containing 0.2 M HBr (Sinopharm Chemical, 40%) and 1.0 M H_2_SO_4_ was used as the anolyte. Both electrolytes were deaerated with flowing argon gas during the experiment. Linear sweep voltammetry was performed using a potentiostat (Iviumstat, Ivium Technologies) at a scan rate of 10 mV s^−1^ under illumination. The simulated AM 1.5-G solar light irradiation (100 mW cm^−2^, Newport Sol 3A, Class AAA Solar simulator) was used as the light source. Chronoamperometry measurements were carried out at various biases under light irradiation.

### SRFC device assembly

The SRFC consists of two electrolyte tanks, a p/n photoelectrolysis cell and a common flow battery component. The p/n photoelectrolysis cell was built inside a home-made reactor with quartz windows. The dual absorbers composed of photocanode and photoanode were placed side by side in cathodic and anodic compartments, respectively. Solution of 0.05 M AQDS+1.0 M H_2_SO_4_ and 0.2 M HBr+1.0 M H_2_SO_4_ was used as initial catholyte and anolyte, respectively. The electrolytes were isolated by a Nafion 115 membrane. During photocharging under AM 1.5-G 100 mW cm^−2^ illumination, the photoelectrodes were directly short-circuited without external bias. The flow rates of the electrolytes were kept at 15 ml min^−1^. After the photocharge, the produced AQDSH_2_ and Br_3_^−^ could either be stored in two isolate tanks or flow through the battery to convert the chemical energy to electricity. In the discharge process, the SRFC was performed at a constant current of 0.5 mA cm^−2^.

### SRFC cycling behaviour

Solutions of 0.05 M AQDS+1.0 M H_2_SO_4_ and 0.2 M HBr+0.005 M Br_2_+1.0 M H_2_SO_4_ were used as initial catholyte and anolyte, respectively. The individual volume of catholyte and anolyte was 2.5 ml. Before photocharge, galvanostatic charge/discharge of the SRFC was performed under dark condition to remove the dissolved oxygen in the electrolytes, preventing the oxidation of the photogenerated AQDSH_2_ species. Photocharge of the SRFC in the first 10 cycles was conducted by directly connecting the photoelectrodes without external bias under visible light illumination (*λ*>420 nm, 100 mW cm^−2^). After the photocharge, the discharge of the SRFC was performed at a constant current of 2.0 mA cm^−2^ under dark condition. The flow rates of the electrolytes were kept at 100 ml min^−1^.

### Calculations

The H-STC energy conversion efficiency (*η*_H-STC_) of photoelectrode can be estimated according to the following equation (assuming that the Faraday efficiency is 100%) (ref. 36):


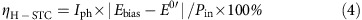


where *I*_ph_ represents the photocurrent density of the photoelectrode, *E*_bias_ stands for the applied bias, *E*^0^*′* is the reversible potential of the redox couple and *P*_in_ denotes the incident solar power (100 mW cm^−2^).

The expected overall STC (*η*_O-STC_) efficiency in the PEC cell can be estimated according to^5^,


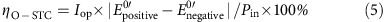


where *I*_op_ stands for the operating photocurrent density calculated based on the total exposure areas of the photoelectrodes, *E*^0^*′* is the reversible potential of the redox couple and *P*_in_ denotes incident solar power (100 mW cm^−2^).

The average STC energy conversion efficiency during photocharge in SRFC system could be roughly estimated according to:


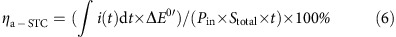


where *i(t)* represents the photocurrent, Δ*E*^0^*′* (V) denotes the potential difference of the two redox couples at reversible state, *P*_in_ is the incident solar power (100 mW cm^−2^), *S*_total_ stands for the total illumination area of p-Si and n-Si, and *t* is the illumination time.

The overall solar(photon)–chemical–electricity energy conversion efficiency during the whole photocharge and discharge process in SRFC system could be calculated according to:


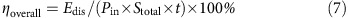


where *E*_dis_ represents the discharged electrical energy, *P*_in_ denotes the incident solar power (100 mW cm^−2^), *S*_total_ stands for the total illumination area of p-Si and n-Si, and *t* is the illumination time.

## Additional information

**How to cite this article:** Liao, S. *et al*. Integrating a dual-silicon photoelectrochemical cell into a redox flow battery for unassisted photocharging. *Nat. Commun.* 7:11474 doi: 10.1038/ncomms11474 (2016).

## Supplementary Material

Supplementary Information Supplementary Figures 1-15, Supplementary Table 1, Supplementary Notes 1-2, Supplementary Methods and Supplementary References.

Supplementary Movie 1 Electric fan powered by electricity generated from as-fabricated SRFC.
