# Supplementary material for: Integrating a dual-silicon photoelectrochemical cell into a redox flow battery for unassisted photocharging
Source: Nat Commun. 2016 May 4;7:11474. doi: 10.1038/ncomms11474 (PMC4857481; doi:10.1038/ncomms11474)
Supplement: Supplementary Information — Supplementary Figures 1-15, Supplementary Table 1, Supplementary Notes 1-2, Supplementary Methods and Supplementary References. [file ncomms11474-s1.pdf]

Supplementary Figures

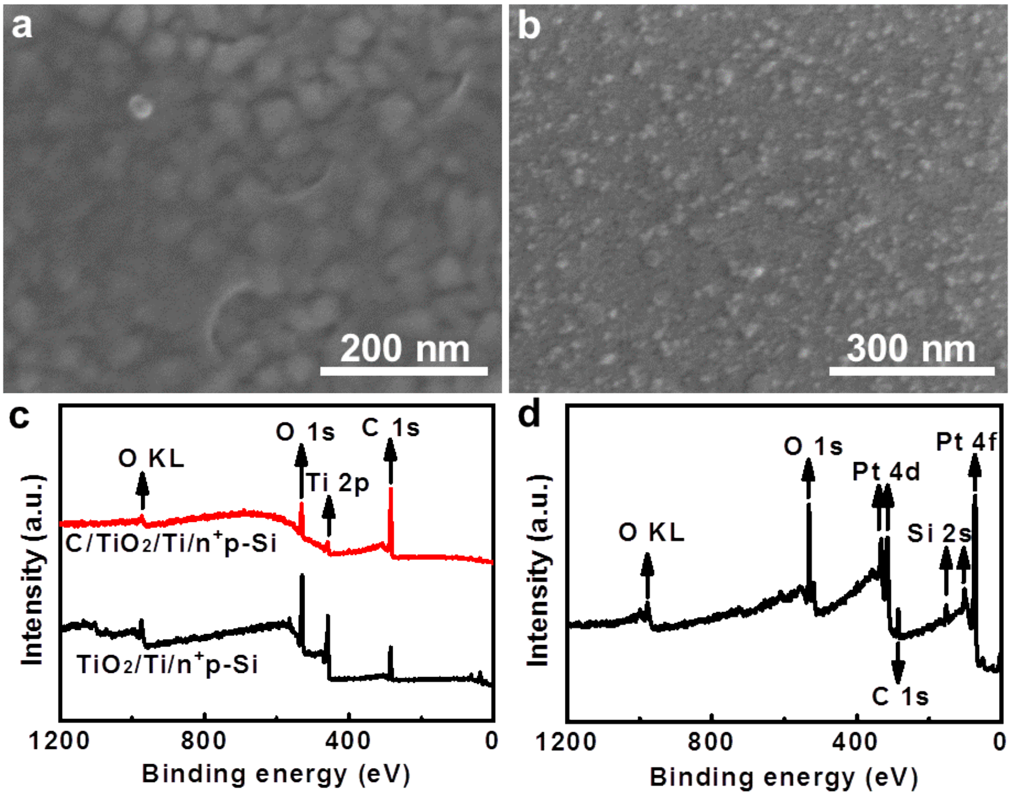

Supplementary Figure 1 | Characterizations of the photoelectrodes. (a) HR-SEM image of the C/TiO_2/Ti/n^+p-Si photocathode. (b) SEM image of the Pt/p^+n-Si photoanode. (c) XPS wide scan spectra of TiO_2/Ti/n^+p-Si and C/TiO_2/Ti/n^+p-Si. (d) XPS wide scan spectra of Pt/p^+n-Si.

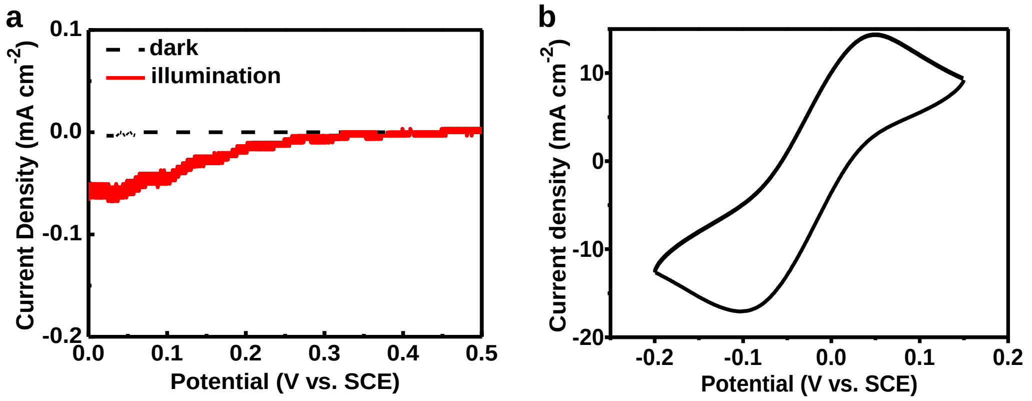

Supplementary Figure 2 | Electrochemical behaviours of various electrodes. (a) Current–potential curves of the C/TiO_2/Ti/n^+p-Si photocathode in a 1.0 M H_2SO_4 solution. Light source: AM 1.5 G 100 mW cm^{-2}. (b) Cyclic voltammogram of 0.05 M AQDS in 1.0 M H_2SO_4 on a graphite electrode.

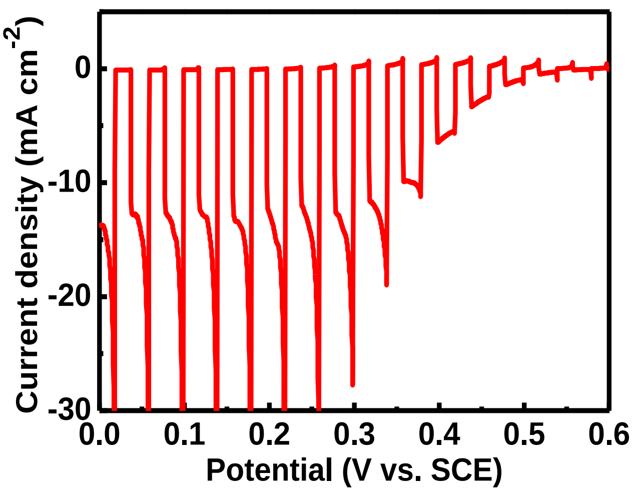

Supplementary Figure 3 | Photoelectrochemical performance of AQDS over C/TiO_2/Ti/n^+p-Si photocathode under the chopped light. Current–potential curves of the photocathodes in a 0.05 M AQDS + 1.0 M H_2SO_4 solution purged with argon under AM 1.5 G 100 mW cm^{-2} illumination.

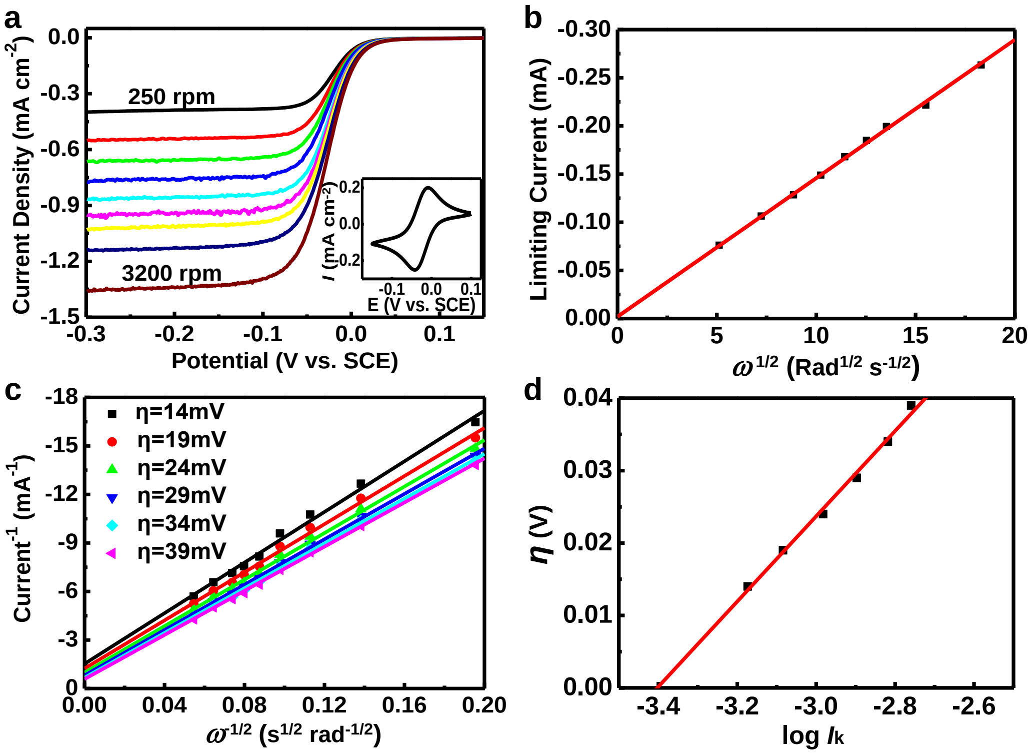

Supplementary Figure 4 | Determinations of kinetic parameter and diffusion coefficient. (a) Rotating disk electrode (RDE) measurements of 1.0 mM AQDS using a glassy carbon electrode in 1.0 M H_2SO_4 at various rotation rates ranging from 250 rpm to 3,200 rpm. Inset in Supplementary Fig. 4a: cyclic voltammogram of 1.0 mM AQDS in 1.0 M H_2SO_4 on a glassy carbon electrode (scan rate 25 mV s^{-2}). (b) Levich plot (limiting current obtained from Supplementary Fig. 4(a) versus square root of rotation rate $\omega^{1/2}$) of 1.0 mM AQDS in 1.0 M H_2SO_4. (c) Koutecky´–Levich plot (i^{-1} versus $\omega^{-1/2}$) derived from Supplementary Fig. 4a at different AQDS reduction overpotentials (η). (d) Fit of Butler-Volmer equation. Constructed using the current response in the absence of mass transport at low overpotentials for AQDS reduction reaction; I_k is the current extrapolated from the zero-intercept of curves in Supplementary Fig. 4c.

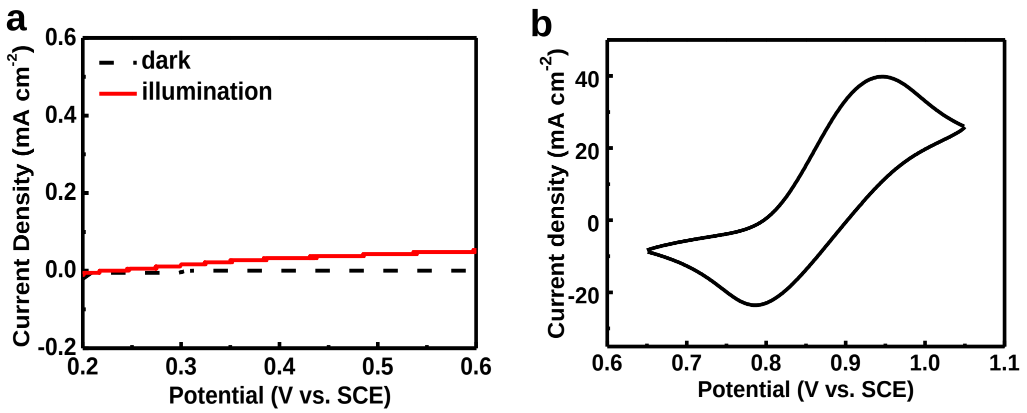

Supplementary Figure 5 | Electrochemical behaviours of various electrodes. (a) Current–potential curves of the Pt/p^+n-Si photoanode in a 1.0 M H_2SO_4 solution. The curves were measured under AM 1.5 G 100 mW cm^{-2} illumination. (b) Cyclic voltammogram of 0.2 M HBr in 1.0 M H_2SO_4 on a Pt electrode.

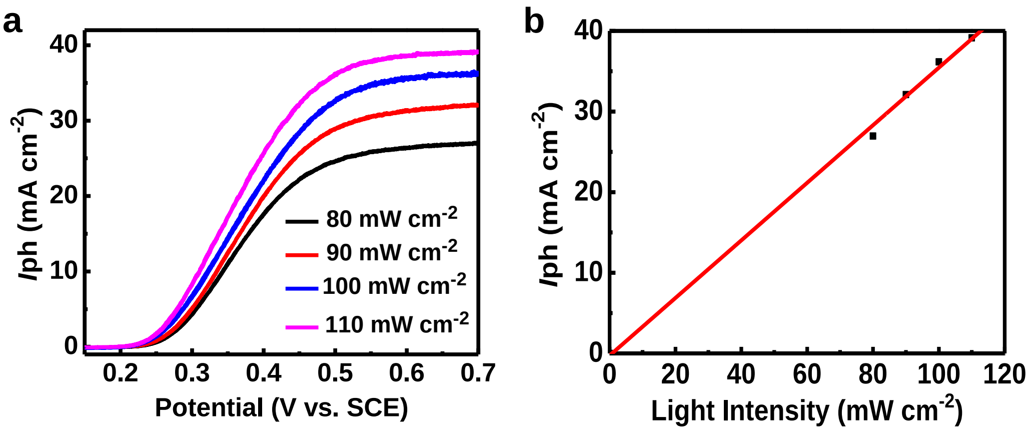

Supplementary Figure 6 | Photoresponse of Pt/p$^+$n-Si under different light intensities. (a) Current–potential curves of the Pt/p$^+$n-Si photoanode in a 0.2 M HBr + 1.0 M H_2SO_4 solution at 600 rpm magnetic stirring speed under various irradiation intensity (AM 1.5 G). (b) Saturation photocurrent density as a function of incident light intensity.

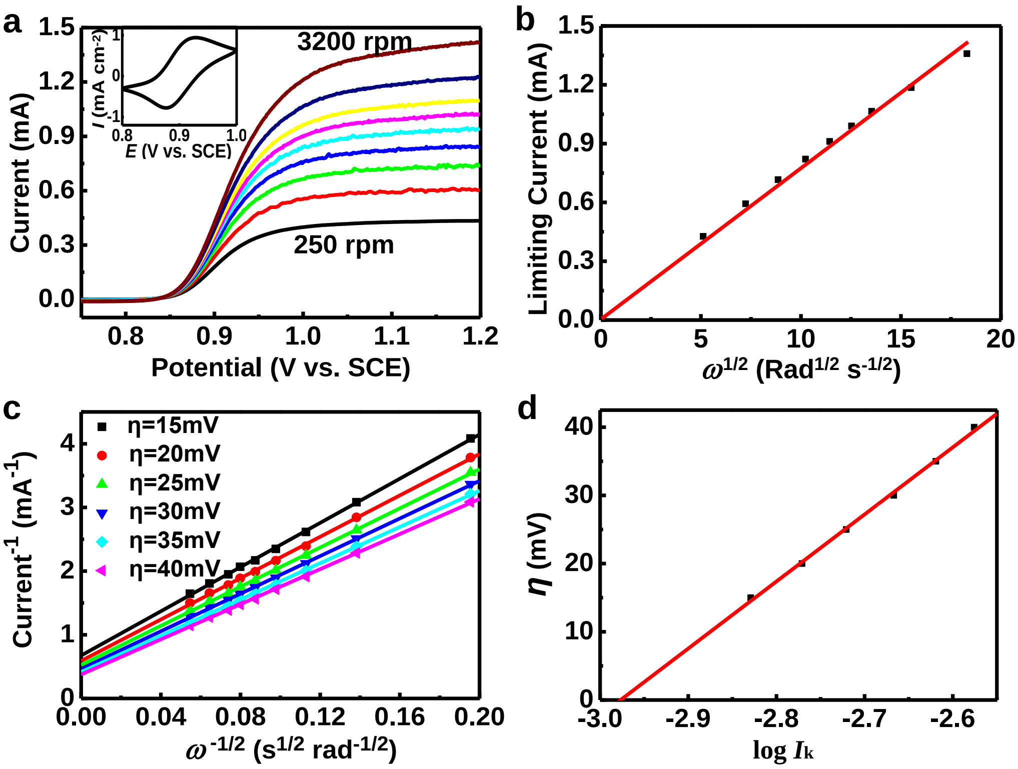

Supplementary Figure 7 | Kinetic parameter and diffusion coefficient determinations. (a) Rotating disk electrode (RDE) measurements of 1.0 mM HBr using a Pt electrode in 1.0 M H_2SO_4 at various rotation rates ranging from 250 rpm to 3,200 rpm. Inset in Supplementary Fig. 7a: cyclic voltammogram of 1.0 mM HBr in 1.0 M H_2SO_4 on a Pt electrode (scan rate 25 mV s^{-2}). (b) Levich plot (limiting current obtained from Supplementary Fig. 7a versus square root of rotation rate $\omega^{1/2}$) of 1.0 mM HBr in 1.0 M H_2SO_4. (c) Koutecky´–Levich plot (i^{-1} versus $\omega^{-1/2}$) derived from Supplementary Fig. 7a at different HBr oxidation overpotentials (η). (d) Fit of Butler-Volmer equation. Constructed using the current response in the absence of mass transport at low HBr oxidation overpotentials; I_k is the current extrapolated from the zero-intercept of Supplementary Fig. 7c.

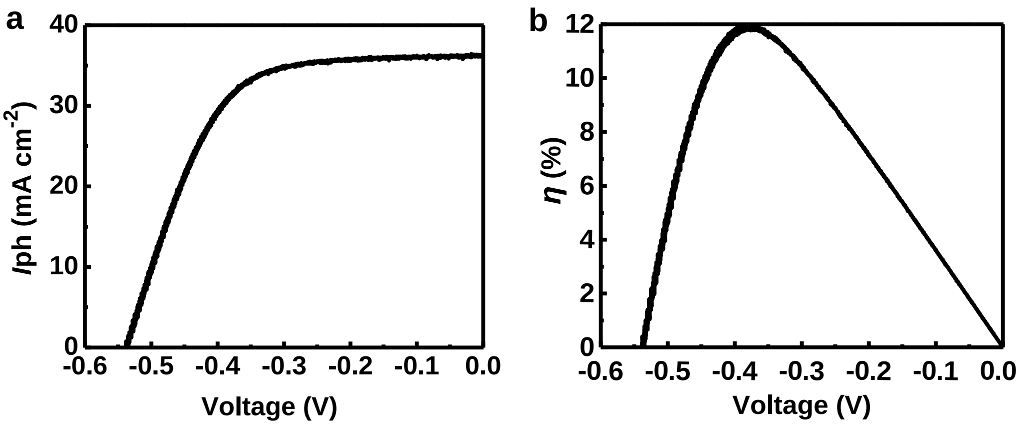

Supplementary Figure 8 | Performance of electrochemical photovoltaic cell. (a) Current−Voltage curve of the Pt/p^+n-Si photoanode in a 6.8 M HBr + 0.1 M Br_2 electrolyte under AM 1.5 G 100 mW cm^{-2} illumination. The measurement was carried out in a two-electrode system consisted of a photoelectrode as the working electrode and a platinum plate as the counter electrode. (b) Corresponding solar-to-electricity conversion efficiency for Pt/p^+n-Si photoanode.

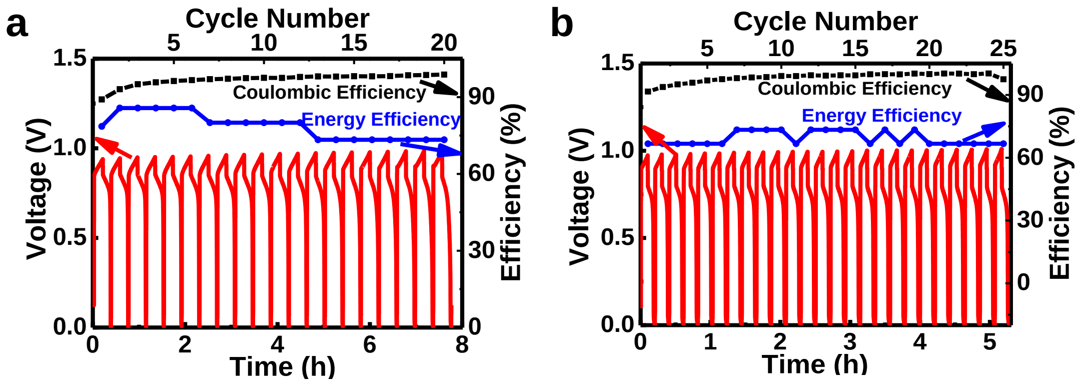

Supplementary Figure 9 | Cycling stability of the RFB. Constant-current cycling at 50.0 mA cm^{-2} (a) and 100.0 mA cm^{-2} (b) at 313 K using a 0.2 M HBr + 0.05 M Br_2 + 1.0 M H_2SO_4 solution on the positive side and a 0.05 M AQDS+1.0 M H_2SO_4 solution on the negative side. The flowing rates of the electrolytes are kept at 130 mL min^{-1}.

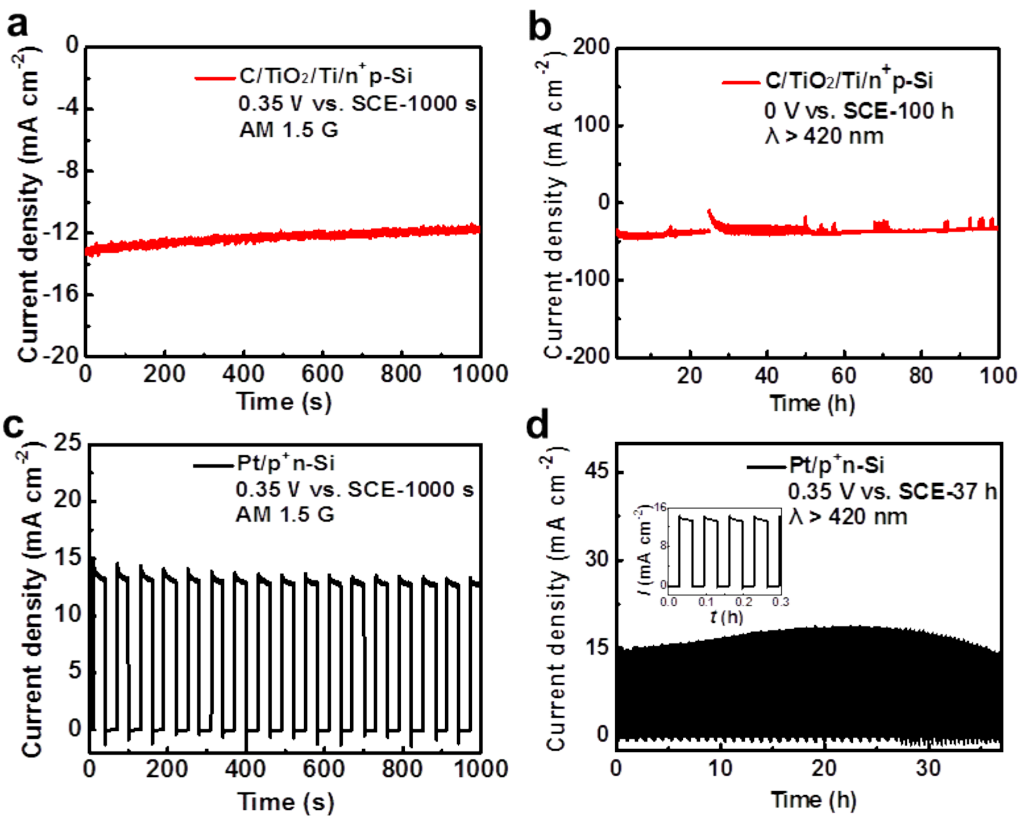

Supplementary Figure 10 | Stabilities of various photoelectrodes under different conditions. Current–time curves of photocathode C/TiO_2/Ti/n^+p-Si in a 0.05 M AQDS + 1.0 M H_2SO_4 solution at 1000 rpm stirring speed under AM 1.5 G 100 mW cm^{-2} illumination (a) and λ > 420 nm, 100 mW cm^{-2} illumination (b). Chopped light current–time curves of the photoanode Pt/p^+n-Si in a 0.2 M HBr + 1.0 M H_2SO_4 solution at 600 rpm stirring speed under AM 1.5 G 100 mW cm^{-2} illumination (c) and λ > 420 nm, 100 mW cm^{-2} illumination (d). The inset in Supplementary Fig. 10d shows chopped light current–time curve of the photoanode in the initial 0.3 h.

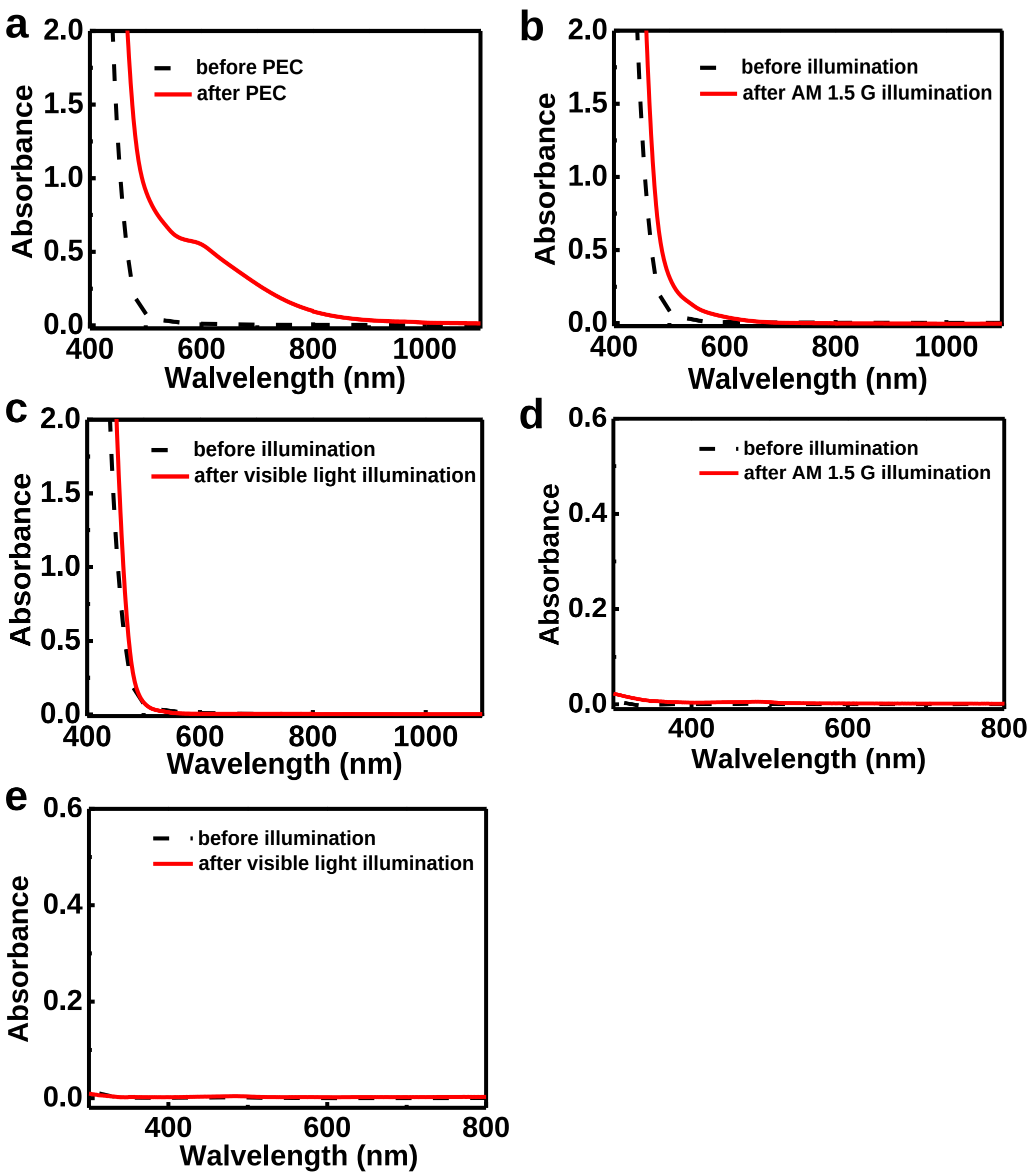

Supplementary Figure 11 | Light absorption of the electrolyte. (a) 0.05M AQDS+1.0M H_2SO_4 solution before and after PEC test at a bias of 0.2 V *vs.* SCE for 10 h under AM 1.5 G 100 mW cm^{-2} illumination. (b) 0.05 M AQDS + 1.0 M H_2SO_4 solution before and after suffering illumination for 8 h under AM 1.5 G 100 mW cm^{-2} illumination. (c) 0.05 M AQDS + 1.0 M H_2SO_4 solution before and after suffering illumination for 13 h under $\lambda > 420$nm, 100 mW cm^{-2} illumination. 0.2 M HBr + 1.0 M H_2SO_4 solution before and after suffering illumination for 10 h under AM 1.5 G 100 mW cm^{-2} illumination (d) and $\lambda > 420$nm, 100 mW cm^{-2} illumination (e). The solutions were de-aerated and kept under a blanket of argon gas throughout the entire experiments.

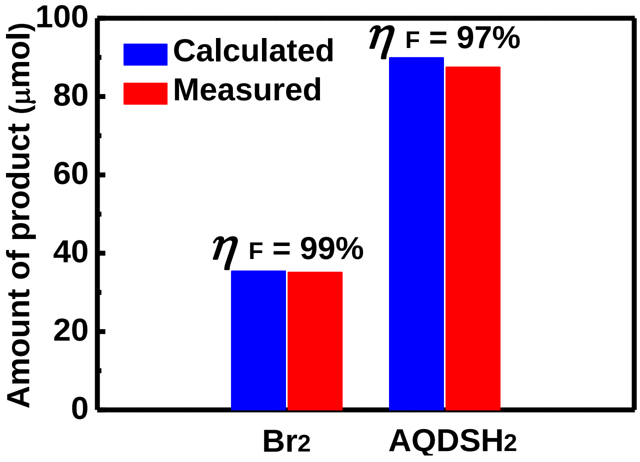

Supplementary Figure 12 | Measurement of Faradaic efficiency. PEC reactions are conducted at the operating potential (0.35 V *vs.* SCE) under visible light illumination.

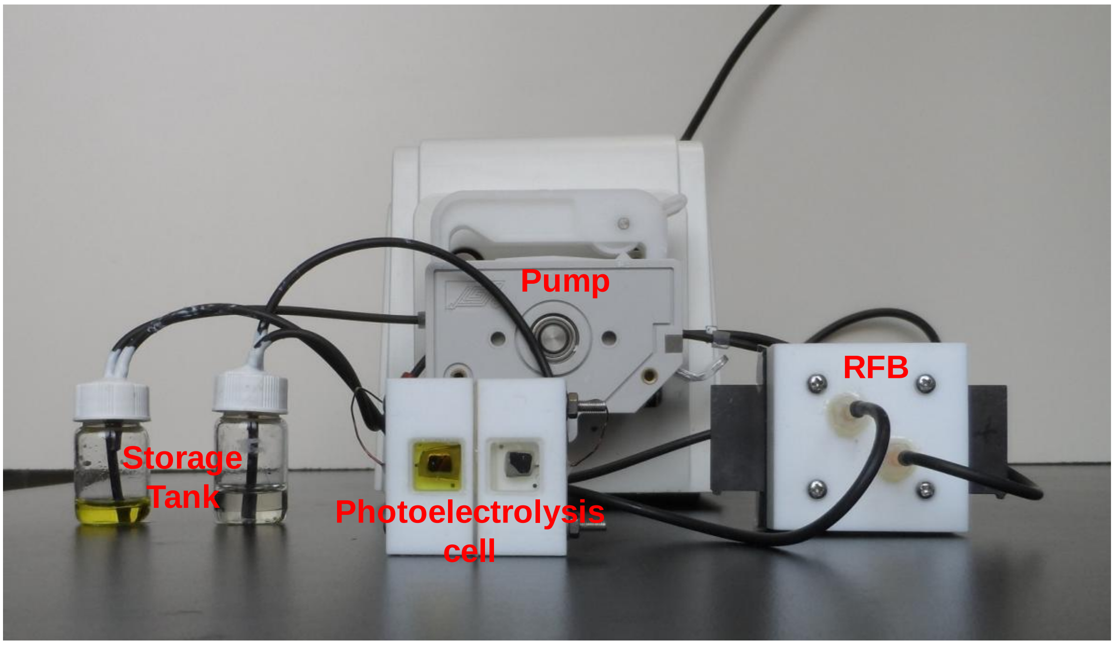

Supplementary Figure 13 | Digital picture of the fabricated proof-of-concept SRFC device. The SRFC contains three parts, two electrolyte tanks, photoelectrolysis cell and battery. The anodic compartment in the photoelectrolysis cell and the positive compartment in the battery are connected to the Br_3^-/Br^- tanks by electrolyte circuit loops, while the other compartment in the photoelectrolysis cell and battery are linked to the AQDS/$AQDSH_2$ tanks. One photoanode and one photocathode are employed in photocharge process and placed in the anodic and cathodic compartment respectively in the photoelectrolysis cell, while two conventional carbon paper electrodes are used as the positive electrode and negative electrode respectively for electrochemical discharging and placed in the positive and negative compartment in the battery.

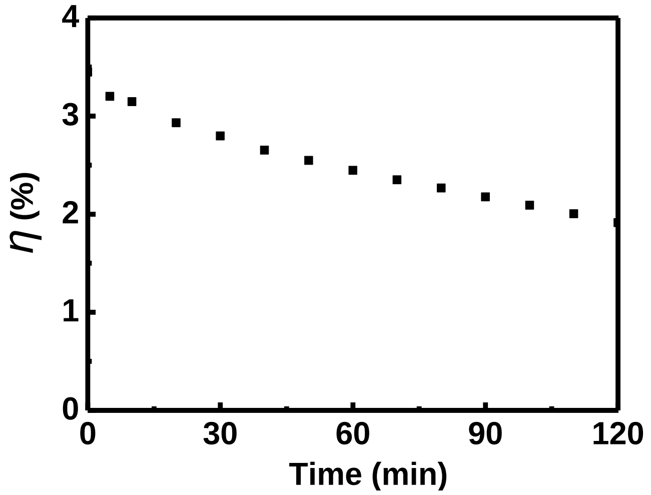

Supplementary Figure 14 | Plots of calculated solar-to-chemical conversion efficiencies versus photo-charge time.

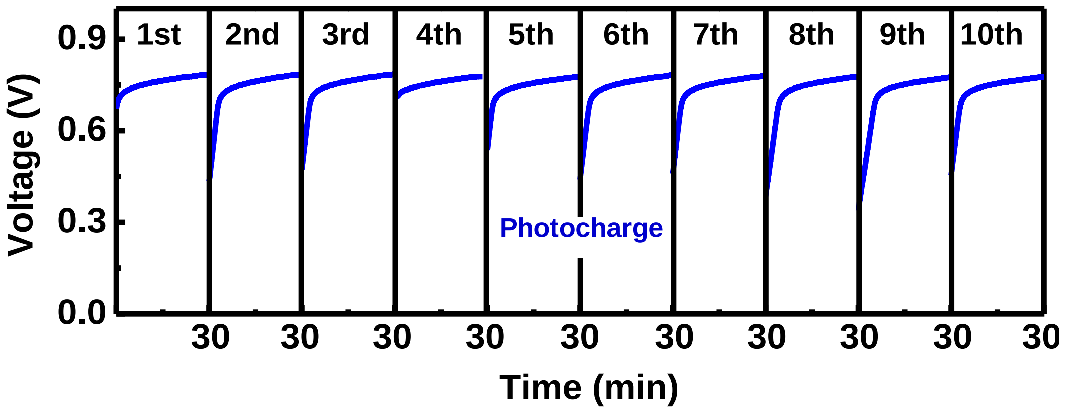

Supplementary Figure 15 | Voltage curves of the SRFC during the 30 min photocharge process for the first 10 cycles.

Supplementary Table 1 | Kinetic parameters for common redox reactions[1-3].

Redox couple	k_0 (cm s^{-1})	Electrode	D (cm^2 s^{-1})	Reference
Fe^{3+}/Fe^{2+}	1.2×10^{-5}	Au(111)	5.8×10^{-6}	1
Cr^{3+}/Cr^{2+}	2.0×10^{-4}	Hg		2
Ce^{4+}/Ce^{3+}	1.6×10^{-3}	Pt		2
VO_2^{+}/VO^{2+}	7.5×10^{-4}	GC	1.4×10^{-6}	3
V^{3+}/V^{2+}	1.7×10^{-5}	GC	1.4×10^{-6}	3
Br_3^{-}/Br^{-}	2.82×10^{-2}	Pt	6.0×10^{-5}	This work
$AQDS/AQDSH_2$	1.05×10^{-2}	GC	4.8×10^{-6}	This work

Supplementary Notes

Supplementary Note 1 | Photoelectrochemical performance of AQDS over $C/TiO_2/Ti/n^+p$-Si photocathode under the chopped light.

The *I–E* curve under the chopped light is usually used to evaluate the utilization of photogenerated carriers that reach the photoelectrode surface. Over the entire potential range in Supplementary Fig. 3, the photocurrent response is characteristic of the decay from a "spike" to a steady state upon light on. Interestingly, the lengths of the photocurrent spikes remarkably increase with the negative shift of applied potentials until the transient currents are up to about –30.0 mA cm^{-2}, where they quickly decay to a constant value of about –12.5 mA cm^{-2} at imposed potentials ≤ 0.25 V *vs.* SCE. This behavior is quite different from those common sluggish reactions like PEC water oxidation[4]. Considering the fast kinetics of AQDS reduction reaction, it is speculated that these instantaneous currents decay at more negative biases might be resulted from the recombination of photogenerated holes and unconsumed photogenerated electrons due to the rapid depletion of AQDS at the photoelectrode surface. In other words, the inadequate supply of electroactive species limited by the mass transfer from the electrolyte to the surface of the photoelectrode is likely responsible for this unusual PEC behavior.

Supplementary Note 2 | Electrochemical performance of the RFB.

The SRFC in this work is an integrating device composing of a photoelectrolysis cell and a RFB. Accordingly, the overall energy conversion efficiency of a SRFC is jointly determined by the solar-to-chemical conversion efficiency of the photoelectrolysis cell and the chemical-to-electricity efficiency of the RFB. Thus, an efficient RFB is also essential for the overall energy conversion of the SRFC. Based on Br_3^-/Br^- and AQDS/$AQDSH_2$ redox couples in aqueous H_2SO_4 media, we fabricated a RFB with carbon as discharge electrodes and evaluated its electrochemical performance. Supplementary Fig. 9a shows the cycling stability of the battery at a constant current density of ±50 mA cm^{-2}. The cell indicates a Faradaic efficiency around 98% with a round-trip energy efficiency between 73% and 86%. When the cell was cycled at a constant current density of ±100 mA cm^{-2} for 25 cycles, the charge/discharge curves are highly reproducible and present a Faradaic efficiency of *ca*. 95% and an energy efficiency of *ca.* 67% (Supplementary Fig. 9b). These efficiencies are comparable to that for other types of redox flow batteries[5].

Supplementary Methods

Chemicals

In all experiments, analytical grade chemicals were used without further purification. Water used was obtained by purifying deionized water with a Milli-Q water purification system.

Electrodes preparation

The p-Si wafers (B doped, 2.2 Ω cm, and 500 μm thickness) and the modified p-Si wafers were used as photocathodes. To achieve a thin surface n^+ doping, the wafers were doped with phosphorous by a vapor phase surface doping process according to reported procedures[6]. The surface doping was carried out under an atmospheric pressure using $POCl_3$ as the P source. The surface doping was performed at 900 ^oC for 10 minutes whereby a shallow n^+p-juntion was formed in the surface of the p-type wafer. Using UV photolithography a 3 µm deep part of the n^+p-Si was etched away, by reactive ion etching, to form 10 mm diameter circular n^+p-junction area. Using UV photolithography the wafers were patterned with a lift-off mask which would allow deposition on the circular junction areas only. The native oxide was removed by soaking the samples in buffered HF. For the case of the 100 nm TiO_2/5 nm Ti/n^+p Si electrodes, TiO_2 was reactively sputtered at room temperature directly after the Ti sputtering. To improve the catalytic activity toward the AQDS reduction, 5 nm carbon film was sputtered on the surface of the TiO_2/Ti/n^+p-Si. Carbon film modification was

deposited by reactively sputtering of a high-purity graphite target (99.999%) in an Ar (1.5 Pa) atmosphere at 500 ^{o}C with a DC power of 120 W for 30 s. After sputtering, the sample was annealed in vacuum at 500 ^{o}C for 0.5 h.

The n-Si wafers (P doped, 1.0 ~ 20.0 Ωcm, and 500 μm thickness, Topsil) were used as photoanodes. To achieve a thin surface p^+ doping, the clean n-Si wafers were subjected to a pre-deposition process in the same manner as in previous work[7]. The doping was carried out in an atmospheric pressure tube furnace in close proximity with BoronPlus planar diffusion sources (Techneglas, Perrysburg, OH, USA). The wafers were inserted into the furnace at 973 K, and the temperature was then ramped at 10 K min^{-1} to 1223 K where it remained for 10 min before cooling at 10 K min^{-1} back to 973 K where the wafers were extracted. The doping run was performed under 5 standard litre per minute (SLM) N_2(g) and 0.1 SLM O_2(g) during the process. Following the doping run, the boron phase layer was removed by dipping the wafers in buffered hydrofluoric acid (BHF) for 5 min. The dopant on the unpolished side of the wafer was removed simply by etching about 5 μm of the surface away in a Reactive Ion Etching (RIE) process (Pegasus, SPTS Technologies). The circular doped areas on the front of the wafer was created by a simple UV lithography process followed by a 3 μm deep reactive ion etch that left the doped areas as 3 μm tall mesas on the front of the wafer. Then, 5 nm Pt was sputtered on the surface of the modified p^+n-Si to reduce the overpotential associated with the Br^- oxidation.

Characterizations

The morphologies of the electrodes were investigated with a Quanta 200 FEG scanning electron microscope (SEM) equipped with an energy dispersive S3 spectrometer (operating at an accelerating voltage of 20 kV). X-ray photoelectron spectroscopy (XPS) measurements were performed using a Thermo ESCALAB 250Xi system with monochromatized Al Kα excitation.

Electrochemical analysis

Kinetic parameters and diffusion coefficients determinations were conducted in a three-electrode system with a rotating glassy carbon or Pt disk, a platinum wire, and a saturated calomel electrode as the working electrode, counter electrode, reference electrode, respectively. The solutions used in the electrochemical analysis were consisted of 1.0 mM AQDS or 1 mM HBr in 1.0 M H_2SO_4. The 1.0 mM AQDS + 1.0 M H_2SO_4 electrolyte was thoroughly purged with argon gas during the entire experiment. All measurements were performed on CHI 760D electrochemical workstation (Shanghai Chenhua Instruments, China) at a scan rate of 10 mV s^{-1} over a range of rotation rates (250, 500, 750, 1000, 1250, 1500, 1750, 2300, 3200 rpm). Kinetic parameters and diffusion coefficients were calculated according to previous work[8]. The diffusion coefficient could be evaluated from the slope of the limited current versus rotation speed curves. The slope was defined by the Levich equation as $0.620nFAC_OD^{2/3}\upsilon^{-1/6}$, where n = 2, Faraday's constant F = 96,485 C mol^{-1}, electrode area A = 0.19625 cm^2, concentration $C_O = 10^{-6}$ mol cm^{-3}, kinematic viscosity υ = 0.01

cm^2 s^{-1}. The kinetic current (i_k) could be obtained using Koutecký-Levich analysis. A plot of $\log_{10}$ (i_k) versus overpotential was linearly fitted with a slope defined by the Butler–Volmer equation as $2.3\alpha RT/nF$, where R is the universal gas constant, T is temperature in kelvin and α is the charge transfer coefficient. The x-intercept gives the log of the exchange current i_0, which is equal to $nFAC_Ok_0$. Cyclic voltammetry was carried out on a static glassy carbon or platinum disk at a scan rate of 25 mV s^{-1}.

Chemical assay of the photogenerated species and quantification of Faradaic efficiency

The light absorption of the electrolytes was analyzed with UV-visible absorption spectroscopy on an UV-Vis-NIR spectrophotometer (Cary 5000). The Faradaic efficiencies for oxidation and reduction half reaction during photocharge are calculated based upon the real amount of photogenerated Br_2 and $AQDSH_2$ and that calculated from chronoamperometry test, respectively. The produced Br_2 during PEC oxidation was firstly extracted with CCl_4, and then the CCl_4 phase was analyzed with UV-visible absorption spectroscopy. The generated $AQDSH_2$ during PEC reduction was detected through electrochemical discharge of the battery.

RFB device assemble

A RFB with aqueous solutions of 0.2 M HBr + 0.05 M Br_2 + 1.0 M H_2SO_4 as anolyte and 0.05 M AQDS+1.0 M H_2SO_4 as catholyte was constructed according to the reported protocols[8]. Graphite plates with interdigitated flow channels (channel width

1.0 mm, channel depth 1.0 mm, landing between channels 1.0 mm) were used as current collectors. Pretreated, stacked (×3) Toray carbon paper electrodes were used on both sides of the cell. DuPont Nafion membrane was used as a proton-exchange membrane and PTFE gasketing was used to seal the cell assembly. The solutions were deaerated before each measurement. Charge/discharge studies of the flow redox battery were carried out under a constant current density of 50 mA cm^{-2} and 100 mA cm^{-2}, respectively. The electrochemical measurements were made to run at constant current for a given period of time, so that 60% of the capacity of the battery was used in each cycle. The flowing rates of the electrolytes were kept at 130 mL min^{-1} and the operating temperature was kept at 313 K.

Supplementary Reference

1 Hromadova, M. & Fawcett, W. R. Studies of double-layer effects at single crystal gold electrodes II. The reduction kinetics of hexaaquairon(III) ion in aqueous solutions. *J. Phys. Chem. A* **105**, 104-111 (2001).

2 Weber, A. Z. *et al.* Redox flow batteries: a review. *J. Appl. Electrochem.* **41**, 1137-1164 (2011).

3 Ding, C., Zhang, H. M., Li, X. F., Liu, T. & Xing, F. Vanadium flow battery for energy storage: prospects and challenges. *J. Phys. Chem. Lett.* **4**, 1281-1294 (2013).

4 Klahr, B., Gimenez, S., Fabregat-Santiago, F., Bisquert, J. & Hamann, T. W. Electrochemical and photoelectrochemical investigation of water oxidation with hematite electrodes. *Energy Environ. Sci.* **5**, 7626-7636 (2012).

5 Skyllas-Kazacos, M., Chakrabarti, M., Hajimolana, S., Mjalli, F. & Saleem, M. Progress in flow battery research and development. *J. Electrochem. Soc.* **158**, R55-R79 (2011).

6 Seger, B. *et al.* Using TiO_2 as a conductive protective layer for photocathodic H_2 evolution. *J. Am. Chem. Soc.* **135**, 1057-1064 (2013).

7 Mei, B. *et al.* Protection of p^+n-Si photoanodes by sputter-deposited Ir/IrO_x thin films. *J. Phys. Chem. Lett.* **5**, 1948-1952 (2014).

8 Huskinson, B. *et al.* A metal-free organic-inorganic aqueous flow battery. *Nature* **505**, 195-198 (2014).
